# Health-related behaviour in Europe – A comparison of selected indicators for Germany and the European Union

**DOI:** 10.17886/RKI-GBE-2017-037

**Published:** 2017-06-14

**Authors:** Cornelia Lange, Jonas D. Finger

**Affiliations:** Robert Koch Institute, Department for Epidemiology and Health Monitoring, Berlin, Germany

**Keywords:** OBESITY, DIET, PHYSICAL ACTIVITY, SUBSTANCE USE, EUROPEAN COMPARISON

## Abstract

Demographic change, new health threats, but also inequalities in health and health care provision in and between European Union (EU) member states pose major albeit similar challenges to European health systems. Regular information on health and health-related behaviour is essential if member states’ health systems are to respond and develop appropriately to these challenges. The ‘European Health Interview Survey’ (EHIS) is a vital source of data for indicators of health status and health-related behaviour in the EU.

This article presents a comparative review of health-related behaviour at the European level. Health-related behaviour is of particular relevance because an unhealthy diet, physical inactivity, obesity, smoking, and harmful use of alcohol are among the most important determinants associated with non-communicable chronic diseases. Eurostat has used data from EHIS Wave 2 to publish details about the current prevalence of obesity, daily fruit and vegetable intake, health-enhancing aerobic physical activity, smoking and heavy episodic drinking for the EU’s member states. In the following, the figures for Germany are compared to the European average. A wide range of prevalences exists between the various EU member states, in some cases stretching to more than 50 percentage points.

In Germany, the prevalence of obesity and smoking remains relatively close to the EU average. Moreover, the results on physical activity are especially welcome. In particular, the proportion of women and men who undertake adequate levels of physical activity decreases more slowly with increasing age compared to the EU average. Nevertheless, the low fruit and vegetable intake, especially among younger generations, and the high proportion of women and men who drink six or more alcoholic beverages on one occasion (heavy episodic drinking) at least once a month pose problems for Germany.

In summary, the results provided by EHIS offer a basis for sharing experiences between EU member states regarding effective measures in health promotion and disease prevention.

## 1. Introduction

### 1.1 Health in Europe

Demographic change, new health threats, but also inequalities in health and healthcare provision in and between European Union (EU) member states pose major albeit similar challenges to European health systems. The European Health Strategy ‘Together for Health’ underpins the EU’s overall Europe 2020 strategy [1]. The European Health Strategy serves as a starting point for action at the national and EU level and complements member states’ health policies. It focuses on increasing cooperation and coordination between member states and developing solutions to the challenges faced by European health systems. In addition, investment in a comprehensive, high-quality healthcare programme and in programmes that promote health is aimed at reducing inequalities and combating social exclusion [2]. If member states’ health systems are to respond and develop appropriately, regular information on developments in living conditions, health, health-related behaviour and the healthcare provision offered to the people of Europe is essential. In the future, the existing activities and tools that provide pan-European information on health are to be broadened as part of a European health information system. This includes further developing the European Core Indicators for Health (ECHI) [3] and ensuring they are increasingly implemented within member states’ health systems [4].

### 1.2 Health-related behaviour - selected aspects and public health relevance

This article provides a comparative review of health-related behaviour in Europe using data from the ‘European Health Interview Survey’ (EHIS). Health-related behaviour is particularly relevant because an unhealthy diet, physical inactivity, obesity, smoking and harmful use of alcohol are among the most important factors associated with non-communicable chronic diseases (NCDs) [5].

The World Health Organization (WHO) estimates that 80% of cardiovascular diseases and strokes, 80% of type 2 diabetes, and 40% of cancer cases could be prevented by following a healthy diet, engaging in sufficient physical activity and non-smoking [6, 7].

According to recent estimates in the 2015 Global Burden of Disease Study, up to 73% of ischaemic heart diseases, 52% of strokes, 84% of lung cancer cases, 55% of colorectal cancer cases, 70% of chronic obstructive pulmonary diseases, 47% of diabetes cases, and 12% of depressive disorders could be avoided by reducing modifiable behavioural risk factors [8]. Despite the improvements in health-related behaviour in Western Europe between 1990 and 2015 which slightly reduced the prevention potential in the field of behavioural risk factors for preventing ischaemic heart disease, stroke, and lung cancer [8, 9], the figures set out above clearly demonstrate that further action is still required.

The WHO’s Global Action Plan for the Prevention and Control of NCDs 2013-2020 takes into account a number of modifiable risk factors. The Action Plan is aimed at ensuring the following targets are met by 2025 in comparison with 2010: a 30% relative reduction in tobacco use, a 30% relative reduction in salt intake, a 10% relative reduction in prevalence of insufficient physical activity, a 10% reduction in harmful use of alcohol, and halt the rise in diabetes and obesity [10]. In order to achieve these targets, health-promoting living conditions are to be created to enable people to live a healthier lifestyle. The Global Strategy on Diet, Physical Activity and Health [11], the WHO European Strategy for Smoking Cessation Policy [12], and the Global Strategy to Reduce Harmful Use of Alcohol [13] are key pillars for meeting the aims that underpin the Action Plan.

The risk factors described above are not only individually problematic; combined, they have a substantial impact on quality of life, healthy aging and mortality [14]. Longitudinal studies convincingly demonstrate that non-smoking, sufficient physical activity, ensuring an adequate intake of fruit and vegetables and moderate alcohol consumption contribute to a better quality of life [15], a healthier aging process, a reduced risk of stroke [17], and a lower risk of mortality by providing up to 14 extra years of life [18, 19].

## 2. Methodology

In addition to official statistics, routine data, and issue-specific international reporting systems (such as on accidents or drug use), data from surveys are a key source of information for European health indicators. In accordance with EU Regulation 1338/2008 on community statistics on public health and health and safety at work, the European Health Interview Survey (EHIS) (see Health monitoring and health indicators in Europe) is an essential source of data for indicators of health and health-related behaviour. EHIS is to be carried out every five years. The first EHIS wave was conducted between 2006 and 2009, but member states were not obliged to participate in the study at this time. Germany integrated a set of selected EHIS questions into the ‘German Health Update’ (GEDA 2010), which was conducted by the Robert Koch Institute [21]. Data on selected health indicators were delivered to the Statistical Office of the European Union (Eurostat).

The second wave of EHIS was prepared as part of a process that was conducted over a number of years. It resulted in the adoption of an EU regulation in February 2013 that specified the variables that need to be collected, the reference year, population, and information about the methodological approach [20]. Eurostat has also prepared a detailed methodological manual containing a model questionnaire [22]. However, as each member state implements EHIS independently, the questions used in the survey are sometimes operationalised differently, and data collection modes can vary (paper, telephone, personal interview). EHIS is divided into four modules: health status, health care, health determinants, and core social variables on demography and socioeconomic status. In Germany, EHIS Wave 2 was integrated into GEDA 2014/2015. In addition to the questions posed for EHIS, further questions that were specific to Germany were asked as part of the survey to enable certain trends to be analysed and to gain insights into other relevant aspects of public health. A description of the methodology applied in GEDA 2014/2015-EHIS can be found in Saß, Lange, and Finger et al. [23]. The methodology applied in EHIS is described in an article by Fehr et al. [2].

Each member state provides the EHIS microdata set to Eurostat. Eurostat uses the quality-assured data it receives to calculate indicators, usually stratifying them according to age, gender, and educational status, before publishing them on its website [24]. This paper focuses on the results of the aggregated data available from the Eurostat database. Therefore, statistical tests for differences cannot be conducted with this data. Moreover, a microdata set that includes results from all countries participating in EHIS Wave 2 will not be available until the end of 2017 at the earliest, when it will be provided by Eurostat on request.

The indicators of health-related behaviour in Europe presented in the following include data from people aged 15 or above, as this reflects the way in which EHIS is implemented throughout the EU. When comparing the prevalences described below with those described in the fact sheets published in this issue of the Journal of Health Monitoring (that use data for Germany from GEDA 2014/2015-EHIS), it is important to remember that the fact sheets focus on data from a slightly different age group: people aged 18 or above. At the same time, different weighting factors were used when analysing data at the European and national levels [25].

## 3. Indicators and results

### 3.1 Obesity

Indicator: Obesity is defined as a large amount of excess weight that results in a body mass index (weight in kg/height in m^2^) of over 30 kg/m^2^. Obesity is a risk factor associated with a number of chronic diseases such as type 2 diabetes mellitus [26], cardiovascular diseases [27] and some forms of cancer [28]. It is also associated with a higher risk of premature death [29, 30]. Obesity and its consequences pose a major challenge to the health system and constitute an important public health problem, not only in Germany but internationally (see Overweight and obesity among adults in Germany). EHIS Wave 2 collected self-reported data on height and body weight. Respondents were asked to state their weight without clothing and their height without shoes, and pregnant women were asked to state their weight before pregnancy.

**Figure 1:**
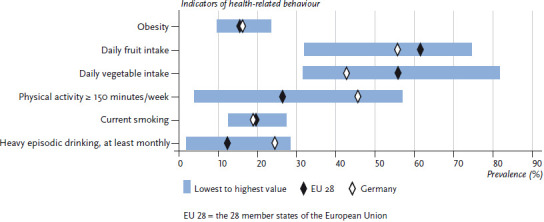
The prevalence of selected indicators of health-related behaviour among women. Data for Germany compared to the average calculated for the EU 28 Data source: EHIS Wave 2

**Figure 2:**
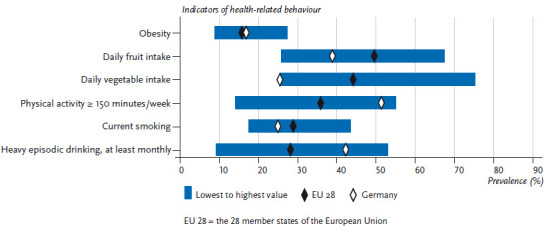
The prevalence of selected indicators of health-related behaviour among men. Data for Germany compared to the average calculated for the EU 28 Data source: EHIS Wave 2

**Figure 3:**
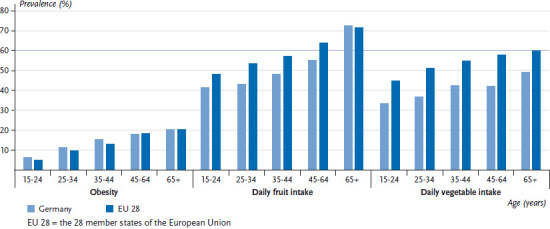
The prevalence of obesity, and fruit and vegetable intake among women by age in Germany compared to the EU 28 Data source: EHIS Wave 2

**Figure 4:**
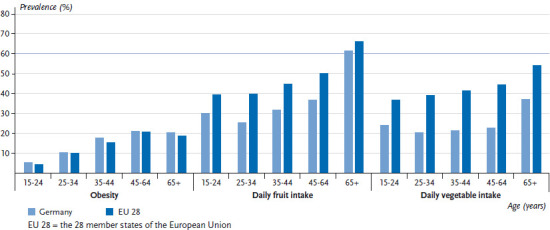
The prevalence of obesity, and fruit and vegetable intake among men by age in Germany compared to the EU 28 Data source: EHIS Wave 2

On average, 15.3% of women and 15.6% of men in the EU aged 15 or above are obese. In Germany, the prevalence of obesity is slightly higher in this age group, with 16.1% of women and 16.7% of men classified as obese. No systematic differences were observed between women and men in terms of obesity prevalence [31]. The prevalence of obesity across the EU ranges from 9.4% to 23.2% among women, and 8.7% to 27.2% among men (Figures 1 and 2). It is impossible to estimate the extent to which these differences are influenced by cultural perceptions of height and body weight, or whether they are indeed reflections of actual differences between countries. However, self-reporting frequently leads to an underestimated body weight and an overestimated height compared to measured values. As a result, body mass indices calculated using self-reported data are generally lower than those based on direct measurements [32]. The prevalence of obesity increases with age. A comparison of the EU average with the prevalence found in Germany according to age demonstrates an above-average prevalence of obesity among women and men in Germany, particularly among younger age groups (to the age of 44). There is virtually no difference between the prevalence observed in Germany and the EU average among people aged 45 and over (Figures 3 and 4).

### 3.2 Daily fruit intake

Indicator: The prevalence of ‘at least daily’ fruit intake. A high intake of fruit and vegetables can help people avoid coronary heart disease, hypertension and stroke, and it can also have a positive impact on the course of these diseases [33-36]. It is also likely that a high fruit intake can help prevent various types of cancer, but the relationship to the overall risk of cancer is low [34, 37-39]. A current meta-analysis has shown that a high fruit and vegetable intake is associated with a lower overall risk of mortality, particularly due to the associated lower risk of cardiovascular mortality [8]. These findings are reflected in recommendations stating that five portions of fruit and vegetables should be eaten daily [40]. Data for the indicator on fruit intake was collected using the question: ‘How often do you eat fruit, including freshly pressed juices?’, with the possible answers ‘Once or more a day’, ‘4 to 6 times a week’, ‘1 to 3 times a week’, ‘Less than once a week’ and ‘Never’ (see Fruit consumption among adults in German and Vegetable consumption among adults in Germany).

On average, 61.5% of women and 49.4% of men in the EU eat fruit at least once a day. Therefore, the figures in Germany (women: 55.6%; men: 38.7%) are below the EU average. Fruit intake in the EU ranges from 31.8% to 74.5% among women and from 25.7% to 67.3% among men (Figures 1 and 2). This places Germany among the bottom third when compared to the EU as a whole. On average, the proportion of women and men who eat fruit at least daily increases with age, both in Germany and in the wider EU. Although the proportion of people under the age of 65 who eat fruit in Germany is well below the EU average, there is no difference between Germany and the EU average when it comes to women aged 65 or above. There is a similar trend among men: the difference between the figures for Germany and the EU average is lowest among people aged 65 or above (Figures 3 and 4). In summary, the figures on EU average fruit intake demonstrate that people are not eating enough fruit to meet the recommendations [41, 42]. Moreover, fruit intake in Germany is even lower than the EU average and urgently needs to be increased; this particularly applies to younger age groups.

### 3.3 Daily vegetable intake

Indicator: The prevalence of ‘at least daily’ vegetable intake was assessed with the question: ‘How often do you eat vegetables or salad, excluding potatoes and juice made from concentrate?’ The following answer categories were provided: ‘Once or more a day’ ‘4 to 6 times a week’, ‘1 to 3 times a week’, ‘Less than once a week’ or ‘Never’.

On average, 55.7% of women and 44.0% of men in the EU eat vegetables at least daily. However, the figures for Germany (women: 42.5%; men: 25.3%) are well below the EU average. In the EU, daily vegetable intake ranges from 31.4% to 81.6% for women and from 25.3% to 75.1% for men (Figures 1 and 2). Men in Germany thus come last in Europe in terms of daily vegetable intake, with women in Germany occupying the fourth from last place in the EU comparison. Moreover, whereas the average proportion of people who eat vegetables at least daily in the EU increases with age, in Germany the proportion of men who eat vegetables daily actually decreases until the 25-to-34 age group before rising again with age. The proportion of women who eat vegetables every day in Germany increases until the 35-to-44 age group but then hardly changes with age. The largest deviation from the EU average among men in Germany occurs among 25-to -64-year-olds: daily vegetable intake by this age group is roughly half of the EU average. A similar tendency is observed among women but it is markedly less pronounced (Figures 3 and 4). Overall, vegetable intake in the EU as a whole remains lower than the recommended levels [41, 42]. Furthermore, a comparison of vegetable intake in Germany with the EU average clearly demonstrates that vegetable intake in Germany is still much lower than the recommended levels, especially among 25-to-64-year-olds. This statement is particularly applicable to men. Therefore, people need to urgently increase the amounts of vegetables that they eat.

According to the Global Database on the Implementation of Nutrition Action (GINA), only a few EU countries have implemented policy strategies and national action plans aimed at encouraging healthy eating [43]. However, in EU countries where relevant action plans and strategies have been put in place, such as France (the French National Nutrition and Health Program [44]) or the United Kingdom (the Eatwell Guide [45]), fruit and vegetable intake is either higher than or roughly equal to the EU average [24]. The aim of the ‘5 a day’ campaign, which recommends that people eat five portions of fruit and vegetables every day [40], is met by just under 10% of people living in Germany; in the EU as a whole, 14% of respondents achieved this aim [46]. In contrast, in the United Kingdom, the Netherlands and Denmark, does at least one quarter of the population reach this target [46].

### 3.4 Physical activity

Indicator: The prevalence of people who meet the WHO’s recommendations on aerobic physical activity (en durance activity) [47]. The WHO recommends that adults should undertake a total of at least 150 minutes of moderate-intensity aerobic physical activity per week (such as cycling, jogging, football, or swimming) that increases breathing and heart rate and continues for at least 10 minutes without interruption [48] (see Health-enhancing physical activity during leisure time among adults in Germany). According to a recent meta-analysis of 80 studies, people with the highest levels of physical activity have around a 35% lower risk of all-cause mortality than those with the lowest level of activity [49]. This indicator was calculated using the version of the European Health Interview Survey – Physical Activity Questionnaire (EHIS-PAQ) that has been validated for Germany [50, 51]. Respondents were asked about the duration of any moderate-intensity aerobic physical activity that they undertake during their leisure time each week (not including work-related activity), including their use of a bicycle for transport.

**Figure 5:**
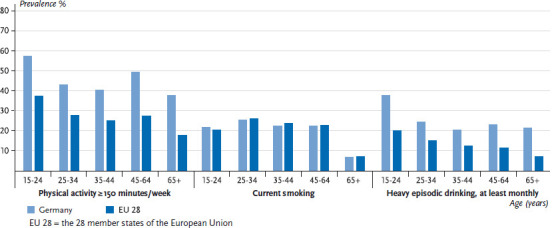
The prevalence of physical activity, smoking and drinking among women by age in Germany compared to the EU 28 Data source: EHIS Wave 2

**Figure 6:**
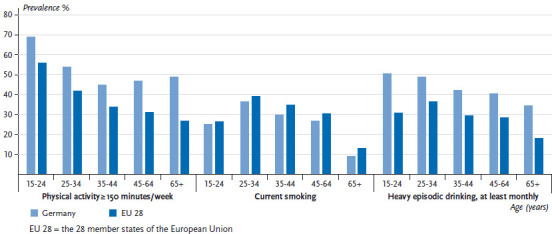
The prevalence of physical activity, smoking and drinking among men by age in Germany compared to the EU 28 Data source: EHIS Wave 2

On average, 26.2% of women and 35.7% of men in the EU meet the WHO’s recommendation of at least 150 minutes of moderate-intensity aerobic activity per week. The levels for Germany (women: 45.5%; men: 51.2%) are above the EU average. In the EU, the prevalence of people who meet the WHO’s recommendations on aerobic physical activity ranges from 3.7% to 56.7% among women and from 14.0% to 54.8% among men (Figures 1 and 2). This places Germany in the top third in the EU comparison. The proportion of people who engage in aerobic physical activity for at least 150 minutes per week is highest in the youngest age group (15 to 24 years of age) and decreases with age. This trend is observed throughout the EU, including in Germany. However, in contrast to the EU trend, men in Germany aged 65 and over have a slightly higher prevalence of meeting the recommendations compared to the 45-to-64 age group. A similar trend is observed among women in Germany, with the difference from the EU average towards a higher prevalence is highest among women aged 65 and over (Figures 5 and 6).

A comparison with the EU average clearly demonstrates that the prevalence in Germany is above average for this indicator. Traditionally, Germany has had an important publicly organised club-based sport sector [52], which could explain the high levels of sports and exercise undertaken in Germany compared to other EU countries. In a current European comparison of policy approaches with regard to club-based sport, it is striking that, in addition to Germany, the Nordic welfare states such as Denmark and Norway in particular have a large publicly organised sports sector [53]. The data from EHIS Wave 2 also show that women in the Scandinavian countries (Sweden, Finland, Norway, and Denmark) are the only ones more likely to reach the recommended levels of aerobic activity than women in Germany [24].

### 3.5 Current smoking

Indicator: The prevalence of current smoking. The ‘current smoking’ indicator includes daily or occasional smoking. Smoking continues to represent one of the most harmful risk factors for a wide range of diseases. According to a recent meta-analysis, smokers have an up to 80% higher risk of all-cause mortality compared to non-smokers. The increased risk continues into old age and follows a dose-response relationship [54] (see Smoking among adults in Germany). Data for this indicator was assessed with the question: ‘Do you smoke?’ (answer categories: ‘Yes, daily’, ‘Yes, occasionally’, ‘No, not any more’, ‘I have never smoked’).

In the EU, the average prevalence of current smoking is 19.5% for women and 28.7% for men. In Germany, smoking rates are below the EU average, both among women (18.8%) and men (24.8%). Current smoking in the EU ranges from 12.3% to 27.2% among women and from 17.4% to 43.3% among men (Figures 1 and 2). This places men in Germany in the bottom third and women in the middle third compared to the EU average. In both Germany and the EU as a whole, the average proportion of people who currently smoke increases until the 25-to-34 age group before decreasing with age. The prevalence of male smokers in all age groups in Germany is significantly lower than the EU average. In contrast, there is no difference between the figures on female smokers in Germany and the EU average in any age group, except for the 15-to-24 age group, where current smoking is actually slightly higher than the EU average (Figures 5 and 6).

Comparing smoking in Germany with the EU average clearly demonstrates that Germany has a comparatively large proportion of smokers and that a further reduction in smoking is needed; this is especially the case with women in the youngest age group. Despite the progress that has been made since 2002 through measures such as increased taxes on tobacco products, stricter age limits on purchasing tobacco products, advertising bans, and laws aimed at protecting non-smokers at the national and federal-state level, there is still room for improvement. In 2016, evaluations made by the Tobacco Control Scale placed Germany second to last (behind Austria). This report compared efforts made by 35 European countries to effectively prevent and control tobacco use [55]. Finally, it remains to be seen whether EU-wide measures aimed at reducing smoking levels, such as the EU Tobacco Products Directive [56], which was to be incorporated into national law by May 2016, will contribute to a change in smoking rates in the EU’s member states.

### 3.6 Heavy episodic drinking

Indicator: The prevalence of heavy episodic (HED) drinking is defined as the consumption of 60 g or more of pure alcohol on a single occasion at least once a month [57]. HED is a particularly harmful pattern of drinking that can cause acute damage such as alcohol poisoning and injuries, and that can also lead to violence. Moreover, in the long term, HED can result in alcohol dependency and a wide range of organic damage. These consequences can even occur if the average level of alcohol consumed is relatively low [57] (see Alcohol consumption among adults in Germany: risky drinking levels). Data was collected for this indicator using the following question: ‘In the past 12 month, how often have you had six or more drinks containing alcohol on one occasion? For instance, during a party, a meal, an evening out with friends, alone at home, …’ The nine possible responses were grouped into four categories: ‘At least every week’, ‘Every month’, ‘Less than once a month’ and ‘Never’. In accordance with the WHO’s definition of HED, this indicator is based on a combination of the categories ‘at least every week’ and ‘every month’, which were combined to form the category ‘at least monthly heavy episodic drinking’.

On average, the prevalence of monthly HED in the EU is 12.2% for women and 28.0% for men. The prevalence of heavy episodic drinkers in Germany, however, is well above the EU average (women: 24.3%; men 42.1%). Throughout the EU, the prevalence of monthly HED range from 1.7% to 28.1% among women and from 9.0% to 52.9% among men (Figures 1 and 2). This places German men in the upper third compared to the EU as a whole. Germany also has the largest prevalence of female heavy episodic drinkers after Denmark. The highest prevalence of heavy episodic drinkers is found among the youngest age group (15 to 24 years of age) among both genders. Whereas on average, the prevalence of female heavy episodic drinkers decreases continuously with age throughout the EU, in Germany the prevalence of HED remains high among women 25 years and above (more than one-fifth of women are heavy episodic drinkers in this age group). Among men in Germany, HED is at its highest in the 15-to-24 age group, but these rates decline among people aged 25 and above. In the EU, the prevalence of heavy episodic drinking among men increases until the 25-to-34 age group and then decreases with age. The prevalence of HED among women in younger age groups in Germany is about twice as high than the EU average and almost three times higher among women aged 65 or above. In the case of men in Germany, the prevalence of heavy episodic drinkers in younger age groups is about 50% higher than the EU average, and about twice as high as the EU average among people aged 65 and above (Figures 5 and 6). The comparison with the EU average clearly demonstrates that HED is comparatively widespread in Germany among all age groups, and that the marked decline that occurs with age in the rest of the EU is not as pronounced in Germany. This result also reflects the fact that far fewer regulatory measures to limit alcohol consumption have been put in place in Germany than in other EU countries [58].

## 4. Discussion and outlook

This comparative review of indicators of health-related behaviour reveals an extremely wide range of prevalences between EU member states. In some cases, the difference constitutes more than 50 percentage points; this is the case with women’s daily vegetable intake and the proportion of women who meet the recommendations on aerobic physical activity (although the differences between Germany and the EU average for current smoking – especially among women – and obesity are comparatively small). The extent to which the (at times very large) differences between individual member states can be explained by different cultural perceptions and answers to the standardised EHIS questions [59], or whether they do in fact demonstrate actual differences in prevalence cannot be answered using the macrodata analysed here and published by Eurostat. Comparing the results with national results obtained from other surveys would also only be partially illuminating, since the results acquired particularly from questions on behaviour, such as fruit and vegetable intake, physical activity and alcohol consumption, vary depending on the type of survey instruments employed. In fact, the data on physical activity and alcohol consumption were assessed using instruments that were especially developed for EHIS [51, 60]. Finally, the fact that the data are linked to specific populations and particular age structures represents a further limitation in terms of comparability. Consequently, before making further comparisons, the data would need to be standardised by age in order to compensate for the different age structures found throughout the EU. Once the microdata set is available for all countries participating in EHIS Wave 2, it will be possible to conduct further cross-sectional analyses that could enable conclusions to be drawn about country-specific response patterns while also taking the different age structures into account.

With these limitations in mind, the results demonstrate that the prevalence of obesity and current smoking in Germany is relatively close to the EU average. The results on physical activity are especially encouraging. In particular, the prevalence of physically active women and men drops significantly less with age in Germany than throughout the EU. This suggests that a considerable proportion of the population in Germany already follows the recommendations on physical activity [61]. However, the very low levels of fruit and vegetable intake, especially among men in the younger age groups, and the very large proportion of female and male heavy episodic drinkers who drink in this manner at least once a month pose a problem. Women in Germany tend to demonstrate a relatively high level of behaviour that has traditionally been attributed to men (such as heavy episodic drinking or physical activity [62]), and in some cases these levels are only surpassed by women from Scandinavian countries. The prevalence of these forms of behaviour among women in Central and Southern European countries is very low.

In closing, the results of the EHIS study can provide a basis for sharing experiences between member states on effective health promotion and prevention measures. EHIS Wave 2 offers the opportunity to use standardised instruments to directly compare the prevalence of health-related behaviour and relate this to the health policies in the respective countries for the first time. Previously, it was only possible to compare frequency distribution patterns and to study basic developments in trends. Moreover, the results provide evidence of the impact of health policy measures. Lastly, EHIS-2 indicators of smoking, obesity, fruit and vegetable intake, physical activity, and heavy episodic drinking are also used alongside other EHIS indicators within the context of the Joint Assessment Framework in the Area of Health (JAFH). This constitutes a first-step screening device aimed at demonstrating the challenges currently faced by health systems in the EU member states [63].

## Key statements

- The proportion of obese women and men in Germany is close to the EU average.
- Germany is in the bottom third compared to the EU as a whole on fruit and vegetable intake, with men in Germany in last place on vegetable intake.
- The population in Germany, especially older people, is more physically active than other EU citizens.
- In Germany, young women smoke more than the EU average; men in all age groups in Germany are less likely to smoke than the EU average.
- Women and men in Germany are close to the top of the list when it comes to heavy episodic drinking in the EU.
